# Health sciences students’ Covid-19 digital health literacy – information reliability evaluation: Nikoleta Leventi

**DOI:** 10.1093/eurpub/ckac130.161

**Published:** 2022-10-25

**Authors:** N Leventi, A Vodenicharova

**Affiliations:** Faculty of Public Health, Medical University - Sofia, Sofia, Bulgaria

## Abstract

**Background:**

In the Shanghai Declaration on promoting health in the 2030 Agenda of the UNs for Sustainable Development highlighted the importance of health literacy (HL) to empower individual citizens and enable their engagement in collective health promotion action. The aim of this study was to demonstrate the COVID-19 digital HL and the information-seeking behavior among students in Medical University - Sofia.

**Methods:**

For the purpose of the study a web-based questionnaire was distributed among medical students from the Faculty of Medicine, and health sciences students from the Faculty of Public Health and the Medical College-Sofia, all from Medical University - Sofia in Bulgaria. The study was conducted between February and April 2022. In total 239 respondents participated, all anonymously and voluntarily. In data analysis established statistical methods were used.

**Results:**

Data collected show that when students searched the Internet for information on the coronavirus or related topics, nearly two thirds (66%) of the respondents could either easy or very easy decide whether the information is reliable or not, and (81%) could easy or very easy decide whether the information is written with commercial interests. In addition among the respondents 82% find easy or very easy to check different websites to see whether they provide the same information.

**Conclusions:**

From the presented analysis the following conclusion can be made: when navigate the social media platforms and forums, it is significant for health sciences students to obtain the appropriate searching skills, in order to be confident to identify the validity of the information and make informed decisions, as well as decide whether commercial interests are in focus of the provided information. Furthermore it is important to emphasize that searching competencies help to cross check the provided information.

**Key messages:**

- In emergency situations like the global COVID-19 pandemic, digital health literacy is an important factor when search and use reliable and crosschecked information in daily clinical practice.
- Health sciences students should be educated aiming to maximize their digital literacy, to acquire the competencies and skills and to be confident when search, and use digitally provided information.

